# Measuring the State Dependence Effect in Hospital Payment Adjustment

**DOI:** 10.3390/ijerph192114110

**Published:** 2022-10-28

**Authors:** Lu Liu, Wei Nai, Zan Yang

**Affiliations:** 1 School of Business, St. Bonaventure University, St. Bonaventure, NY 14778, USA; 2 School of Electronic Information, Huzhou College, Huzhou 313000, China; 3 Public Teaching and Research Department, Huzhou College, Huzhou 313000, China

**Keywords:** health economics, public policy, econometrics, hospital payment adjustment, state dependence effect

## Abstract

Since FY 2013, as a part of the Affordable Care Act (ACA) program, the Hospital Value-Based Purchasing (HVBP) program has adjusted Medicare’s payments to hospitals based on the total performance score of the hospital. First, the program reduces a portion of the hospital’s Medicare payments in a specific fiscal year, and then, by the end of the same fiscal year, the amount of the payment reductions will be awarded to the hospitals based on the total performance score; thus, the hospitals that do not receive the reward will lose the portion of money reduced by Medicare. In this research, we apply the theory of state dependence and use the dynamic random effect probit model to estimate this effect. The results show that the hospital payment adjustment dynamics have a very significant state dependence effect (0.341); this means that hospitals that received a reward in the previous year are 34.1% more likely to receive a reward this year than the ones that received a penalty in the previous year. Meanwhile, we also find that the state dependence effect varies significantly across hospitals with different ownership (proprietary/government owned/voluntary nonprofit), and the results show that voluntary nonprofit hospitals exhibit the largest effect of state dependence (0.370), while government-owned hospitals exhibit the lowest effect of state dependence (0.293), and proprietary hospitals are in the middle. Among the factors that influence the likelihood that a hospital receives a reward, we find that teaching hospitals with a large number of beds (>400) are less likely be rewarded; in terms of ownership, we find that voluntary nonprofit hospitals are more likely be rewarded; in terms of demographic factors, hospitals where the average household income are higher within the region are more likely be rewarded.

## 1. Introduction

The Hospital Value-Based Purchasing (HVBP) program, starting in FY 2013, has been in effect for nine years. It adjusts the Medicare payment reimbursement based on hospitals’ performance on clinical quality, cost efficiency, safety and patient experience measures. Each year, the Center for Medicare and Medicaid Services (CMS) withholds a percentage of payments from each hospital to fund the program and re-distribute the payment based on the performance of the four dimensions. In FY 2013, the withholding percentage was 1%. Since FY 2017, the withholding percentage has been 2%. In FY 2019, a total of approximately USD 1.9 billion in Medicare reimbursement will be re-distributed through the program’s value-based incentive model.

Previous research on this HVBP program has focused on the impact of the program and what are the factors that lead to a hospital being penalized or rewarded, and the results are mixed. Some researchers suggest that, although the program has an effect, the effect is not significant (Figueroa et al. (2016) [1]); other researchers found that the effect is impacted by the hospitals’ own characteristics, and the effect will vary given the different location and ownership of the hospitals (Haley et al. (2015) [2]); considering the extent of the penalty or reward, researchers found positive associations between the penalty magnitude and certain current-year care process improvements (Lee et al. (2020) [3]). However, if those payment adjustments (penalty or reward) only affect the hospital in the current year or if the previous payment adjustments will influence the current year and future payment adjustment remain important questions. Up to date, no study has systematically studied the persistence of the payment adjustment of the program.

The state dependence effect, introduced by Heckman (1981), refers to the phenomenon that the realization of an event affects the probability that the same event occurring in the future [4]. Two sources of this state dependence effect are as follows: the true state dependence effect, which refers to the effect of a past event happened before; and the spurious state dependence effect, which refers to unobserved heterogeneity. It has many applications in topics of unemployment persistence, innovation behavior and welfare reform. The state dependence effect of payment adjustment of VBP (reward or penalty) on hospitals describes the influence of past reward or penalty status on current and future reward and penalty status. Previous literature with respect to the state dependence effect has considered the financial impact of the VBP program, but whether Heckman’s proposed effect exists in the financial re-distribution of the rewards incentives, and if so, is the strength of that effect and what may be the possible policy implications of that effect have not been discussed; thus, this paper serves well to add to the literature.

## 2. Literature Review

Established by Section 1886 of the Social Security Act, the HVBP program is the first national pay for performance (P4P) program implemented and administrated by CMS. The quality of care is evaluated in four domains: safety, efficiency, clinical care and patient experience. The VBP program’s ultimate goal is to improve the health of Medicare beneficiaries by purchasing better care for them at a lower cost. Different measures are used to evaluate the performance on the four domains. Clinical care is evaluated based on measures that show how closely best clinical practices are followed. There are 13 different measures of clinical care quality across various conditions. Patient experience is evaluated based on eight measures: communication with nurses, communication with doctors, responsiveness of hospital staff, pain management, communication about medicines, cleanliness and quietness of the environment and discharge information. Efficiency is measured by cost per Medicare beneficiary.

Since FY 2014, safety measures have been added to the CMS VBP evaluation criteria, based on the mortality rates of acute myocardial infarction, heart failure and pneumonia. Acute care hospitals located in the 50 U.S. states and District of Columbia (excluding Maryland), with at least 10 cases in each clinical care measures and 100 completed Hospital Consumer Assessment of Healthcare Providers and Systems (HCAHPS) surveys are eligible to participate in the CMS VBP program. For hospitals who are eligible, the participation in the program is mandatory.

CMS (2021) assesses the hospital’s performance by comparing the hospital’s achievement points (awarded by comparing a hospital’s rates during the performance period with all hospitals’ rates during the baseline period) and improvement points (awarded by comparing a hospital’s rates during the performance period with the hospital’s self-ratings during the baseline period). The greater of the two (achievement point and improvement point) is used to calculate the total performance score (TPS) [5]. The weight of the four domains is adjusted from year to year. In 2016, safety carried the most weight at 40%, followed by patient experience and efficiency, which carried 25% weight each. Clinical care accounted for just 10%. In 2020, the weight of the four dimensions will be evenly distributed, with each accounting for 25%.

The program will first reduce a portion of the hospital’s Medicare payments and then distribute this portion of money to the hospitals based on the quality of care provided to patients. For FY 2013, the portion is 1% of total Medicare payments, and the percent will increase by 0.25 each subsequent year. For FY 2017 and later, the portion is set at 2%. In terms of total amount of money distributed by the program, for FY 2013, it is USD 963 million and for FY 2017, it is USD 1.8 billion.

### 2.1. Effectiveness of the VBP Program

Since the introduction of the CMS VBP program, it has aroused a lot of attention both from practitioners and researchers about the impact of the program. However, the results are mixed, and the impact on different perspectives of the program are different.

Figueroa et al. (2016) studied the impact of VBP program on patient mortality of three conditions (acute myocardial infraction, heart failure and pneumonia) using a total of 4267 acute care hospitals in US, among which 1348 were not eligible to participate in the VBP program (critical access hospitals that usually located in rural area and hospitals in Maryland that adopted an all-payer model). He found that, for the hospitals that were impacted by the program, the mortality rates decreased by 0.13%, and for the hospitals that were not impacted by the program, the mortality rates decreased by 0.14% [6]. The difference between the mortality trend of the impacted and the nonimpacted hospitals was not significant. He concluded that there is a lack of evidence that the VBP program will lead to a lower mortality rate, and he suggested alternative models to achieve a lower mortality rate.

Bonfrer et al. (2018) did an observational study comparing hospitals that volunteered to participate in the Premier Hospital Quality Incentive Demonstration (PHQID, the pilot program of VBP) and the hospitals whose incentives were implemented later in the VBP program. The sample of the study included 214 hospitals that had been impacted by the PHQID program since 2003 and 975 matched hospitals that had been impacted by the VBP program since 2013. Their results showed that early adopters and late adopters of the program did not differ significantly in terms of clinical quality or mortality. They concluded that being impacted for a longer time in the program was not likely to make the hospitals perform better [7].

Since the research on the effects of the VBP program largely shows no or little improvements on hospital performance, researchers began to investigate why the VBP program is not effective. Markovitz et al. (2017) reviewed the literature to assess whether area factors, organizational and structural factors play a role in hospital performance. Their results showed that hospitals are not responding strategically to the incentives of the VBP program, and the VBP program needs to increase the financial incentive while at the same time clarifying the incentive structure. They also suggested that, although some heterogeneity across organization types may mask the main effect of the program, the variation is not sufficient enough to alter the conclusion that the VBP program does not meet its original goal [8]. Feng et al. (2019) studied the pay for performance program in England; they found that the program suffered from the complexity of specialized care, which requires the significant specific investments, including linking the performance measures to an evidence base; the complexity also makes the development of health outcome measures more difficult, justifying a focus on process measures and rewarding providers for improvements [9]. Kairies-Schwarz et al. (2020) studied the pay for performance program in Germany hospitals, and their results suggest that the introduction of a pay for performance program has a positive effect on quality of care depending on the monetary incentives, type of participants and the patient orientation [10]. Moscelli et al. (2021) investigated the effect of treatment reform policy from 2002 to 2019 on the quality of hospitals applying a difference-in-difference strategy. Their results found that public hospitals facing more rivals had reduced quality, increased waiting times and reduced length of stay. They concluded that this is due to the regulated price reform [11].

### 2.2. Financial Impact of the VBP Program

While the effectiveness of the VBP program has been studied largely, the attention on the financial impact of the VBP program is lacking. In 2013, the Government Accountability Office (GAO) published a report and documented the changes in payment to the hospitals in the first year of the CMS VBP program. Their report showed that all the 3455 acute care hospitals were impacted by the payment adjustment. They also showed that hospitals in Massachusetts received the largest increase in payments (USD 275 million) and hospitals in five states received a decline of payments over USD 20 million (NY, TX, FL, IL and MI) [12].

The probability of getting penalized by the CMS VBP program is also different across different types of hospitals. Gilman et al. (2015) argued that the VBP program put safety-net hospitals at a financial disadvantage compared with others, and the program had a disproportionate effect on safety-net hospitals. Their results showed that, among all the hospitals, 63% of safety-net hospitals received a penalty in 2014, compared with 51% of other hospitals [13].

Fos (2017) further analyzed this disparity between safety-net hospitals and others in the CMS VBP payment adjustment; in his analysis, he showed that the safety-net hospitals are more likely to be penalized. Further, he performed an analysis on demographic factors and showed that the reason why safety-net hospitals are more likely be penalized is related to the patients, since the patients of safety-net hospitals are mostly low income, uninsured and medically vulnerable. He argued that the payment adjustment structure of CMS VBP will lead to a situation that the hospitals that are serving poor populations are more vulnerable for the penalty [14].

Chen et al. (2017) compared the financial performance of the hospitals in Mississippi Delta Region and non-Delta hospitals under the CMS VBP program. They used a seven-year panel dataset, and a difference-in-difference framework was applied to examine the difference between the Delta and non-Delta hospitals in the pre- and post-period of the VBP program. Their results showed that the Delta hospitals suffered from a 4.24% reduction in operating margin after the initiation of the VBP program, while the non-Delta hospitals achieved a 1% increase in the operating margin. They concluded that, after the implementation of the VBP program, the disparity between the Delta and non-Delta hospitals in financial performance became wider, and they suggested that policy makers should modify the program to make sure the financial situations of the resource unfavored hospitals do not become worse [15].

Liao et al. (2020) studied the effect of nationwide Merit-Based Incentive Payment System (MIPS), proposed by CMS, aiming to incentivize clinicians to improve health care value for Medicare beneficiaries. The MIPS is a pay-for-performance program that increases payment rates for clinicians who provide high-value care, while penalizing clinicians who do not by decreasing their payment rates. Their result suggested that safety-net practices may perform more poorly than their non-safety-net counterparts. Policymakers should monitor for such dynamics and consider ways to adjust MIPS policy to ensure these practices are not inappropriately penalized by the program [16].

### 2.3. State Dependence Effect

The state dependence effect, proposed by Heckman (1981), refers to the phenomenon that the realization of an event affects the probability of the same event occurring in the future, which can be caused by two reasons. The first explanation is that, through experiencing a past event, a certain behavior, for example, preference of a consumer or R&D investment of a firm, is altered. In this explanation, the past experience has a genuine behavioral effect that will lead the individual to behave differently as opposed to the same individual who has not experienced that event. Heckman termed this as “true state dependence” or “structural state dependence”. The second explanation is that individuals may differ in unobserved factors (for example, lack of motivation or low level of capability) that affect their likelihood of experiencing an event (that has nothing to do with whether an individual has experienced that event in the past or not). Heckman termed this as “spurious state dependence”. In his paper, he also proposed a model to distinguish between the true state dependence and spurious state dependence [4].

Heckman’s paper has aroused a lot of attention in economics, finance, health care and other areas. Researchers studied this effect in labor force participation, unemployment persistence and poverty/low pay persistence (Lynch (1985) [17], Gebhard et al. (1993) [18], Arulampalam et al. (2000) [19]), persistence of R&D investment (Flaig et al. (1994) [20], Cefis et al. (2001) [21], Peters (2009) [22], Triguero et al. (2013) [23], Arque-Castells (2013) [24]), dynamics of health (Contoyannis et al. (2004) [25], Halliday (2008) [26], Contoyannis et al. (2011) [27] and Roy et al. (2013) [28]).

In this research, we used a panel with 2471 hospitals impacted by the CMS VBP Program from year 2013 to 2018, with a total of 14,826 observations. The Wooldridge (2005) approach is applied here to help with disentangle from the true state dependence effect and the spurious state dependence effect [29]. We found that the hospital payment adjustment dynamics have a very significant true state dependence effect (0.341), which suggests that hospitals that received a reward in a previous year are 34.1% more likely to receive a reward this year than the ones that received a penalty in a previous year. The persistence due to the state dependence effect explains 77.1% of the persistence observed in the data, and the unobserved heterogeneity explains the other 22.9% of the persistence. Meanwhile, we also found that the state dependence effect varies significantly across hospitals with different ownership (proprietary/government-owned/voluntary nonprofit hospitals), and the results show that voluntary nonprofit hospitals exhibit the largest effect of state dependence (0.370), while government-owned hospitals exhibit the lowest effect of state dependence (0.293), and proprietary hospitals are in the middle.

## 3. Model and Estimator

Given the state dependence effect mentioned above, the state dependence effect in CMS hospital payment adjustment remains unexplored, and the research on the CMS VBP program is mostly about the effectiveness of the program and how it impacts hospital performance. The effect can be modeled as follows:

(1) $$y_{it}={x^{\prime }}_{it}\beta +\gamma y_{ij-1}+\alpha_{i}+u_{it}$$

where ***y****_it_* is the binary outcome of whether a hospital receives a reward (equals 1) or a penalty (equals 0), ***y****_it_*_−1_ is whether the hospital received a reward or penalty in the previous year (lag of dependent variable), ***x****′_it_* is the vector of observed hospital characteristics, ***α****_i_* captures the unobserved heterogeneity and ***u****_it_* is the error term. The null is there is no state dependence (***γ*** = 0). The estimate of parameter ***γ*** is the average state dependence over time, which is our focus.

Several assumptions are contained in the equation. First, the dynamics are first-order dynamics, i.e., ***y****_ij_*_−2_ does not have an effect on ***y****_ij_*; second, ***x****_it_* is appropriately strictly exogenous and conditional on unobserved heterogeneity. The assumptions are the same as Wooldridge (2005) [29].

Given the two assumptions (dynamics are first order; ***x****_it_* is strict exogenous), let ***f***_t_(***y****_t_*|***x****_t_*, ***y****_t_*_−1_, ***α***; ***β***) be the correctly specified density, then the density of (***y****_i_*_1_, …, ***y****_iT_*) is

(2) $$\prod_{i=1}^{N}\prod_{t=1}^{T}f_{t}(y_{it}|x_{it},y_{it-1},\alpha_{i};\beta_{0})$$

To get an estimate of parameter ***β***, we need to face the fact that it depends on the unobservable variable, ***α****_i_*. To solve this, we can treat ***α****_i_* as a parameter to be estimated; this leads to the maximization of the log likelihood function

(3) $$\sum_{i=1}^{N}\sum_{t=1}^{T}\log f_{t}(y_{it}|x_{it},y_{it-1},\alpha_{i};\beta )$$

As is pointed out by Hsiao (1986), the initial conditions will not be a problem if *T* is large; unfortunately, in our dataset, compared with *i*, *T* is small. So, we need to endogenize and model the initial condition to obtain a consistent estimate [30].

In previous research, three ways have been proposed to solve the problem of handling the initial conditions in dynamic nonlinear models, as is summarized by Hsiao (1986). The first one is to treat the initial conditions for each unit as nonrandom; however, this requires very strong assumptions that the initial condition ***y****_i_*_0_ is independent of unobserved heterogeneity. The second approach, proposed by Hsiao (1986), is to use the joint distribution of outcomes on the response condition on unobserved heterogeneity and observed variables and allow for the initial condition to be random. The main difficulty in this approach is to specify the distribution of the initial condition based on unobserved heterogeneity [30]. The last one is to approximate the conditional distribution of the initial condition, as proposed by Heckman (1981), but it is more difficult computationally to obtain an estimate of the parameters and average effects [4].

Here, we apply the Wooldridge (2005) approach to handle this problem [29], which is to model the distribution of unobserved heterogeneity that is conditional on the observed exogenous variables, and the initial values (use the density of (***y****_i_*_1_, …, ***y****_iT_*) that are conditional on (***y****_i_*_0_, ***x****_i_*), i.e., specifying ***f***(***α***|***y****_i_*_0_, ***x****_i_*)). Under this approach, assume ***h***(***c***|***y***_0_, ***z***; ***δ***) is a correctly specified model for the density of ***D***(***c****_i_*|***y****_i_*_0_, ***z****_i_*); then, the density of (***y****_i_*_1_, …, ***y****_iT_*) given (***y****_i_*_0_ = ***y***_0_, ***x****_i_* = ***x***) is:

(4) $$\int_{R^{J}}\left(\prod_{t=1}^{T}f_{t}(y_{t}|x_{t},y_{t-1},\alpha ;\beta_{0})\right)h(\alpha |y_{0},x;\delta_{0})\eta (d\alpha )$$

This leads the log-likelihood function, which is conditional on (***y****_i_*_0_, ***x****_i_*), to be:

(5) $$l_{i}(\beta ,\delta )=\log\left[\int_{R^{J}}\left(\prod_{t=1}^{T}f_{t}(y_{t}|x_{t},y_{t-1},\alpha ;\beta )\right)h(\alpha |y_{i0},x_{i};\delta )\eta (d\alpha )\right]$$

After this, we sum up the log-likelihood function with respect to *i* = 1, …, *N* and maximize it with respect to ***β***, ***δ***, and we get the estimate of ***β***_0_, ***δ***_0_. The resulting conditional MLE is $\sqrt{N}$, which is a consistent and asymptotic normal under standard regularity conditions.

To obtain the estimate of partial effect, let ***q***(***y****_t_*) be a scalar function of ***y****_t_*, then the average partial effects across the distribution of ***α****_i_* is:

(6) $$\mu (x_{t},y_{t-1})=E\left[m(x_{t},y_{t-1},\alpha_{i};\beta_{0})\right]$$

where

(7) $$\begin{array}{ll} m(x_{t},y_{t-1},\alpha_{i};\beta_{0}) & =E\left[q(y_{it})|x_{it}=x_{t},y_{i,t-1}=y_{t-1},\alpha_{i}=\alpha \right]\\ & =\int_{R^{G}}q(y_{t})f_{t}(y_{t}|x_{t},y_{t-1},\alpha ;\beta_{0})v(dy_{t}) \end{array}$$

A consistent estimator can be obtained by

(8) $$\hat{\mu }(x_{t},y_{t-1})=N^{-1}\sum_{i=1}^{N}r(x_{t},y_{t-1},x_{i},y_{i0};\hat{\beta },\hat{\delta })$$

where ***r***(***x****_t_*, ***y****_t_*_−1_, ***x****_i_*, ***y****_i_*_0_; ***β***_0_, ***δ***_0_) = *E*[***m***(***x****_t_*, ***y****_t_*_−1_, ***α****_i_*; ***β***_0_)|***y****_i_*_0_; ***x****_i_*].

The entry probability is

(9) $$e_{it}\equiv Pr(y_{it}=1|y_{it-1}=0,x_{it})=\Phi [(x^{\prime }it\beta ){\left(1-\rho \right)}^{0.5}]$$

The persistence probability is

(10) $$s_{it}=Pr(y_{it}=1|y_{it-1}=1,x_{it})=\Phi [(\gamma +{x^{\prime }}_{it}\beta ){\left(1-\rho \right)}^{0.5}]$$

where **Φ**[] is the standard normal cumulative distribution function and ***ρ*** is the fraction of variance that attributes to the variation in the time-invariant individual effects.

By comparing the raw persistence and predicted persistence, we can derive the percentage of the raw persistence explained by the state dependence effect, which is

(11) $$\frac{Pr(y_{it}=1|y_{it-1}=1,x_{it})-Pr(y_{it}=1|y_{it-1}=0,x_{it})}{Pr(y_{it}=1|y_{it-1}=1)-Pr(y_{it}=1|y_{it-1}=0)}=0$$

## 4. Data and Description

Our data are obtained from three main sources: characteristics of hospitals (for example number of employees, number of beds, number of discharge) are obtained from CMS Impact File, payment adjustment data come from Hospital Inpatient Prospective Payment System (IPPS) and demographics data within a 10-mile radius come from the Census Bureau.

The number of hospitals participating in the program, average adjustment factor and number of hospitals that received an award/penalty are shown in Table 1.

The total number of hospitals varies from year to year because CMS has established a minimum data requirement for the number of cases, measures, surveys, etc. For the patient experience domain, hospitals must report at least 100 patient surveys in order to receive a score for this domain. For the clinical quality and safety measures, hospitals must report a minimum of 10 cases per measure. This number was established through an analysis conducted by Brandeis University and RAND Corporation. In this analysis, CMS sought to balance the need for statistically reliable scores with the policy goal of including as many hospitals as possible in the Hospital VBP Program. Inclusion of data that do not meet the requirement could skew the results and further impact the calculation of the total performance score.

CMS does not publish the exact amount of money that is awarded or penalized for each hospital; they only published the distribution in FY 2016, as seen in Table 2 and Table 3.

Our data comprise a panel with 2471 hospitals from year 2013 to 2018, with a total of 14,826 observations. As is mentioned in the theory part of state dependence, there are two reasons that the realization of an event affects the probability that the same event will occur again in the future. The first one is that a past experience has a genuine behavioral effect that will lead the hospital to behave differently as opposed to the same hospital that has not experienced that event (i.e., true state dependence). The second one is that individuals may differ in unobserved factors (unobserved heterogeneity or spurious state dependence). We plotted the payment adjustment factors for hospitals in Georgia from 2013 to 2018 based on the first year’s adjustment status (rewarded/not rewarded); if visually we do not observe a difference in the pattern, maybe it is a sign that we should not endogenize the initial condition in the estimation.

Since we have the data about whether the hospitals receive a reward during the six-year period (2013 to 2018), we can calculate the conditional probabilities that a hospital will receive a reward this year, conditional on last year’s reward status. If there is no difference in the two conditional probabilities, then there is model-free evidence that last year’s reward status has no effect on this year’s status. Table 4 shows the conditional probabilities.

Comparing Columns 3 and 4 of Table 4, we can see that, if in the previous year a hospital got rewarded, then the next year, its probability of receiving a reward again is about twice of the hospital who got a penalty in the previous year. So, we can see that there is a considerable state dependence in hospitals’ payment adjustment by the HVBP program.

In Table 5, we summarize the dependent variable and explanatory variables used in this study, with their mean value and standard error.

## 5. Empirical Results

In Table 6 we show results of estimates based on the simple pooled probit estimator, random effects probit estimator and Wooldridge estimator. The simple pooled probit model ignores the unobserved heterogeneity and the initial condition problem; the random effects probit model considers the unobserved heterogeneity, but again ignores the initial condition problem; they both overestimate the effect and are used as benchmarking models. The hospitals in the category of rural/proprietary/lowest CMI/no teaching/lowest bed capacity of New England are used as the benchmarking categories.

In Table 7 we show the marginal effect estimates. We can find that, on average, the previous year’s penalty/reward status will have 34.1% of the impact on this year’s penalty/reward status. Other significant factors include the hospital’s own characteristics: if the hospital is larger in terms of the number of employees, it is more likely to be rewarded, mainly because it has better resources; if hospital is larger in terms of the number of discharges/beds, it is less likely to be rewarded due to number of patients treated being so large. The teaching status also has a significant effect: if the hospital has many students and residents, it is less likely to be rewarded; in terms of the patients’ demographics, we found that, hospitals located in an area with higher income are more likely to be rewarded; compared to a small urban area, hospitals in a large urban area are less likely to get rewarded. Additionally, the geographic area has a significant effect, suggesting the payment adjustment program should take geographic area into account as well.

The lag of the dependent variable (reward t − 1) is positive significant across the three estimators, suggesting that there is a positive significant state dependence effect.

The preferred model (Wooldridge model) gives an average marginal effect (the state dependence effect) of 0.341, which means that hospitals that received a reward in the previous year are 34.1% more likely to receive a reward this year than the ones that received a penalty in the previous year. Since for the 2471 hospitals observed from 2013 to 2018, only one hospital in one year (2013) was neutral (neither rewarded nor penalized), the variations of the status of being penalized or rewarded are the same, so the penalty state dependence is the same as the reward state dependence. The persistence due to the true state dependence effect explains 77.1% of the persistence observed in the data (Equation (11)), the unobserved heterogeneity explains the remaining 22.9% of the persistence.

For other explanatory variables, some hospital characteristics are significantly associated with the likelihood of receiving a reward from CMS; for example, the number of employees shows a significant positive effect, the number of beds shows a significant negative effect, the teaching status shows a significant negative effect and the percent of Medicare/Medicaid discharge shows a moderate negative effect. Compared with proprietary hospitals, voluntary nonprofit and government-owned hospitals are more likely to receive a reward.

Among demographic variables, we observe a moderate significant negative effect from the Black and Hispanic population variable, household income shows a significant positive effect on the probability of a hospital to receive a reward and competition show a moderate significant positive effect.

For geographic factors, we observe that, compared with hospitals located in rural areas, hospitals located in urban areas are less likely to receive a reward; compared with hospitals located in the New England area, hospitals located in the Mid-Atlantic and West South Central areas show a significantly lower likelihood of receiving a reward; hospitals located in the East South Central area show a moderately significant less likelihood of receiving a reward, while hospitals located in other areas do not show a significant difference.

Above are the estimates from the three estimators based on the whole sample. We controlled for the hospital ownership and located areas with a set of dummy variables. However, this state dependence effect may differ depending on the different ownership and geographic areas. So, we estimated the state dependence effect for hospitals of different ownership and geo location. A test of equality of coefficients was performed to examine if the state dependence effect across different ownership/geographic areas is the same or not. If the state dependence effect is different across those areas, then there is evidence to suggest the policy design across different kinds of ownership and different geographic areas should be different. By performing the Wald test, we obtained the results shown in Table 8:

The test of equality of coefficient shows a chi-2 value of 8.98 and a *p* value of 0.011, which means that the state dependence effect is significantly different between large urban/other urban/rural hospitals.

We further performed a pairwise comparison test to see if the effect is equal between pairs of large urban/other urban, large urban/rural and other urban/rural hospitals, and the results of the chi-2 and *p* value can be found in the last two rows of Table 9; we can see that, among the three pairs, the state dependence effect differs significantly between hospitals located in large urban areas and other urban areas, and it also differs significantly between hospitals located in large urban areas and rural areas; for other pairs, it is not significantly different. 

The test of equality of coefficient shows a chi-2 value of 7.34 and a *p* value of 0.026, which means that the state dependence effect is significantly different across hospitals of different ownership. For the pairwise comparison, we found that the state dependence effect is significantly different between voluntary nonprofit hospitals and proprietary hospitals; for voluntary nonprofit hospitals and government owned hospitals, it is also significantly different, and for the other pairwise comparison, we did not find a significant difference.

To test the effect of different factors on the extent of the penalty/reward, we further used the payment adjustment factor as the dependent variable and ran two regressions, and the results are shown in Table 10. We found that, for the hospitals that get a reward, the case mix index (which indicates the complexity of the situation of the patient) actually plays an important role, and the hospitals that are treating patients with more complex situations will receive less reward; again, hospitals with a larger number of beds/discharges will receive less reward; hospitals with a larger number of employees and serving patients with higher income will receive more reward; hospitals serving more white populations will receive less reward; hospitals located in large urban areas, will receive less reward. The type of geographical area also impacts the extent of the reward.

## 6. Conclusions, Discussion and Future Work

The Hospital Value-Based Purchasing (HVBP) program, launched and administrated by CMS, is the first national-level p4p program for hospitals in the U.S. Although some research suggests moderate to no improvement in hospital quality, how the payment adjustment decision is made and whether the payment adjustment has a long-lasting effect (other than just an immediate effect) on the hospital have not been studied. In this research, we applied a dynamic probit random effects model to analyze the state dependence effect in hospital payment adjustment. We ask the following questions: does a hospital’s payment adjustment status depends on the previous year’s status, and what are the factors that influence the hospital’s likelihood of receiving a reward in this program? The results showed a positive significant state dependence effect across the three different models we estimated and are significant for hospitals located in different geo areas (large urban/other urban/rural) and hospitals of different ownerships (government-owned/voluntary nonprofit/proprietary).

For the factors that impact the likelihood that a hospital will receive a reward from the HVBP program, we found that the number of employees shows a significant positive effect, suggesting that, as the number of employees grows larger, hospitals have more labor resources and can manage to improve upon the quality measures to reach a reward; the number of beds and discharges shows a significant negative effect, suggesting that, as the patient volume gets heavier, hospitals become unable to meet the quality criteria, suggesting there is a potentially a negative network effect. Teaching status shows a significant negative effect, which makes sense because residents in hospitals are still in their training stage and may not be able to perform at the quality level that is required by the program. The percent of Medicare/Medicaid discharges shows a moderate negative effect. Compared with proprietary hospitals, voluntary nonprofit and government-owned hospitals are more likely to receive a reward.

Among demographic variables, we observe a moderate significant negative effect from the Black and Hispanic population variable, household income shows a significant positive effect on the probability of a hospital receive a reward and competition shows a moderate significant positive effect.

For geographic factors, we observe that, compared with hospitals located in rural areas, hospitals located in urban areas are less likely to receive a reward; compared with hospitals located in the New England area, hospitals located in the Mid-Atlantic and West South Central areas show a significantly less likelihood of receiving a reward, the hospitals located in the East South Central area show a moderately significant less likelihood of receiving a reward, while hospitals located in other areas do not show a significant difference.

In FY 2017, an over USD 690 million payment to hospitals was redistributed by the CMS VBP program from the penalized hospitals to the rewarded hospitals, and the goal of this payment adjustment and redistribution was to improve the overall quality of care. Our results showed that the probability of whether a hospital received a reward or penalty depends on many factors, and hospitals that serve the low-income population are more likely be penalized; additionally, hospitals that serve a larger patient volume and have a heavier teaching responsibility are also more likely be penalized. This reward/penalty payment adjustment is highly state dependent, meaning that, once a hospital is penalized, in the following year, it has a significantly higher probability of being penalized again, and the penalty or reward has a long-term effect.

All the above discussions suggest that the CMS VBP program does not meet its original goal to improve the overall quality of hospitals and reward hospitals with a better performance. We suggest that CMS revise the formula of the total performance calculation and adjust for the socio-economic and demographic factors to make sure the program and payment adjustment do not penalize the hospitals that are already in a disadvantaged position.

VBP rewards or penalizes a hospital based on a hospital’s total performance score across four domains: clinical care (13 measures), efficiency (1 measure), safety (3 measures) and patient experience (8 measures). In all, there are 25 measures. Improvement in any one domain would require significant investments of time and money, and improvement on a single measure is unlikely to produce meaningful change in a hospital’s overall score. According to François De Brantes, director of Altarum’s Center for Payment Innovation (CPI), any program designed to drive quality improvement should include just a handful of measures that are tightly related to what the provider can control. Moreover, the budget-neutral structure of the program makes the return on investment uncertain.

The VBP program scores hospitals using “tournament models” (i.e., providers are scored relative to one another)—and not based on clear, absolute and prospectively set performance targets. Hospitals do not know what their performance is until the end of the performance period. Meanwhile, a 2% ceiling on penalties or rewards does not garner much interest, especially in VBP, where payment adjustments average close to 0.5%. When hospitals have a low probability of knowing what the outcome of the investment is and the reward is relatively weak, they have less motivation to respond to the VBP program.

Over the past eight years in the VBP payment adjustment program, more than half (2046) of the hospitals were penalized; however, recent research by Sankaran (2019) found that the penalized hospitals have little or no evidence of improvement in their performance [31]. This is consistent with our findings that hospitals that received a penalty the year before are 34.1% more likely to receive a penalty again the year after. The CMS VBP program initially was designed to urge the hospital to perform better, but the result is not satisfactory. One of the main reasons is, although the administration team of the hospitals put the VBP program as their top priority, the employees in hospitals accept the program differently. In research by Tevis (2014) [32], the researchers found that physicians usually question the necessary knowledge that patients have to report the quality of healthcare and are less willing to make changes according to the VBP program. The VBP program designed over 50 metrics for the hospitals to consider, but few guidelines on how to improve the performance were given; thus, most hospitals still rely on traditional staff training to improve performance, which is not enough. Given the nature of the state dependency effect we found in this research and the fact that the hospitals located in rural areas serving less privileged people have a higher probability of being penalized by CMS, we suggest that CMS should take into account the patients’ demographics and the teaching status and geographic location of the hospital in their algorithms of determining who should be rewarded and penalized so that the hospitals that are already vulnerable but are critical to the whole healthcare system will not be penalized.

## Figures and Tables

**Table 1:** Number of hospitals awarded and penalized.

Year	Number of Hospital Penalized	Number of Hospital Awarded	Total Number of Hospital	Min Adjustment Factor	Max Adjustment Factor
2013	1426	1557	2984	0.991	1.008
2014	1473	1255	2728	0.989	1.007
2015	1375	1714	3089	0.987	1.021
2016	1235	1806	3041	0.983	1.024
2017	1343	1612	2955	0.982	1.032
2018	1211	1597	2808	0.983	1.030

**Table 2:** Distribution of change of payments for FY 2016.

Change of Payment	Number of Hospitals
>USD 150,000	284
USD 120,001 to USD 150,000	103
USD 90,001 to USD 120,000	172
USD 60,001 to USD 90,000	217
USD 30,001 to USD 60,000	366
USD 1 to USD 30,000	652
USD −30,000 to USD −1	391
USD −60,000 to USD −30,001	182
USD −90,000 to USD −60,001	138
USD −120,000 to USD −90,001	98
USD −150,000 to USD −120,001	73
<= −USD 150,000	349

**Table 3:** Distribution of percentage change of payments for FY 2016.

Change of Percentage of Payment	Number of Hospitals
1.0% < x	316
0.9% < x ≤ 1.0%	77
0.8% < x ≤ 0.9%	92
0.7% < x ≤ 0.8%	94
0.6% < x ≤ 0.7%	108
0.5% < x ≤ 0.6%	123
0.4% < x ≤ 0.5%	174
0.3% < x ≤ 0.4%	194
0.2% < x ≤ 0.3%	194
0.1% < x ≤ 0.2%	212
0.0% < x ≤ 0.1%	210
−0.1% < x ≤ 0.0%	222
−0.2% < x ≤ −0.1%	227
−0.3% < x ≤ −0.2%	197
−0.4% < x ≤ −0.3%	162
−0.5% < x ≤ −0.4%	133
−0.6% < x ≤ −0.5%	85
−0.7% < x ≤ −0.6%	105
−0.8% < x ≤ −0.7%	42
−0.9% < x ≤ −0.8%	37
−1.0% < x ≤ −0.9%	13
x ≤ −1.0%	8

**Table 4:** Conditional and unconditional probabilities that a hospital will receive a reward.

Year	Unconditional (*P_it_* = 1)	Awarded at *t* − 1 (*P_it_* = 1|*P_it_*_−1_ = 1)	Penalized at *t* − 1 (*P_it_* = 1|*P_it_*_−1_ = 0)
2014	0.467	0.677	0.246
2015	0.516	0.675	0.377
2016	0.553	0.786	0.285
2017	0.511	0.717	0.257
2018	0.548	0.815	0.268

**Table 5:** Summary of dependent and explanatory variables.

Variable Name	Description	Mean	SD
Dependent Variable			
Reward Status	Whether a hospital was rewarded (binary variable)	0.518	
Explanatory Variable			
Geographic Characteristics			
New England	CT, ME, MA, NH, RI, VT	0.048	
Mid-Atlantic	NJ, NY, PA	0.129	
East North Central	IL, IN, MI, OH, WI	0.174	
West North Central	IA, KS, MN, MO, NE, ND, SD	0.082	
South Atlantic	DE, FL, GA, MD, NC, SC, VA, DC, WV	0.178	
East South Central	AL, KY, MS, TN	0.084	
West South Central	AR, LA, OK, TX	0.120	
Mountain	AZ, CO, ID, MT, NV, NM, UT, WY	0.065	
Pacific	AL, CA, HI, OR, WA	0.140	
Large Urban Area	Hospital located in a large urban area	0.418	
Other Urban Area	Hospital located in other (small) urban area	0.339	
Rural Area	Hospital located in a rural area	0.243	
Demographic Characteristics			
White Population	Number of White residents in the zip code (in thousands)	18.21	10.81
Black Population	Number of Black residents in the zip code (in thousands)	3.21	4.64
Hispanic Population	Number of Hispanic residents in the zip code (in thousands)	4.16	7.43
Household Income	Average household income in the zip code (in thousands)	45.70	19.77
Competition	Number of people per hospital in 10-mile radius of a hospital (in thousands)	5.23	7.11
Hospital Characteristics			
Ownership			
Government Owned	Hospitals owned by government (district/local/state/federal)	0.154	
Voluntary nonprofit	Voluntary nonprofit hospitals owned by churches or other private entities	0.655	
Proprietary	Proprietary hospitals	0.191	
Bed Capacity	Number of beds in a hospital	226.34	191.41
Small	Hospitals with <100 beds	0.251	
Medium	Hospitals with 100 to 399 beds	0.611	
Large	Hospitals with ≥400 beds	0.138	
Teaching Status	Resident-to-bed ratio in a hospital	0.072	0.166
None	Hospitals with no residents	0.636	
Very Minor	Hospitals with resident-to-bed ratio between 0.001 and 0.049	0.109	
Minor	Hospitals with resident-to-bed ratio between 0.050 and 0.249	0.155	
Major	Hospitals with resident-to-bed ratio between 0.250 and 0.599	0.07	
Very Major	Hospitals with resident-to-bed ratio ≥ 0.600	0.03	
Case Mix Index	Diversity, clinical complexity and the need for resources in a hospital	1.539	0.266
Quartile 1	Hospitals with CMI ≤ 1.254	0.131	
Quartile 2	Hospitals with CMI between 1.255 and 1.446	0.255	
Quartile 3	Hospitals with CMI between 1.447 and 1.645	0.298	
Quartile 4	Hospitals with CMI ≥ 1.646	0.316	
Number of Employees	Number of total paid employees in a hospital	1460.53	1861.51
Number of Discharges	Total number of discharges in a year for a hospital	10,986.35	10,541.99
Percent of Medicare/Medicaid Discharge	The ratio of Medicare/Medicaid discharge over total number of discharges	0.470	0.132

**Table 6:** Results of model estimates of state dependency.

	Pooled Probit	RE Probit	Wooldridge
Reward *t* − 1	1.087 (0.025) ***	0.973 (0.033) ***	0.867 (0.035) ***
Reward 0			0.248 (0.035) ***
No. of Employees *t* − 1	0.073 (0.039) ***	0.079 (0.017) ***	0.085 (0.018) ***
No. of Discharges *t* − 1	−0.008 (0.003) *	−0.009 (0.004) *	−0.009 (0.004) *
Percent of Medicare/Medicaid Discharge *t* − 1	0.019 (0.118)	−0.038 (0.135) *	0.074 (0.140) *
Bed Capacity Medium *t* − 1	−0.296 (0.039) ***	−0.331 (0.045) ***	−0.329 (0.047) ***
Bed Capacity Large *t* − 1	−0.421 (0.071) ***	−0.464 (0.082) ***	−0.467 (0.086) ***
CMI Q2 *t* − 1	0.034 (0.045)	0.051 (0.050)	0.042 (0.052)
CMI Q3 *t* − 1	0.021 (0.049)	0.044 (0.056)	0.033 (0.058)
CMI Q4 *t* − 1	0.009 (0.055)	0.034 (0.062)	0.024 (0.064)
Government Owned	0.072 (0.043)	0.075 (0.051)	0.104 (0.054) *
Voluntary Nonprofit	0.209 (0.034) ***	0.226 (0.040) ***	0.260 (0.042) ***
Very Minor Teaching	−0.121 (0.043) **	−0.139 (0.049) **	−0.143 (0.052) **
Minor Teaching	−0.134 (0.039) **	−0.154 (0.045) **	−0.145 (0.047) **
Major Teaching	−0.171 (0.059) **	−0.195 (0.068) **	−0.204 (0.070) **
Very Major Teaching	−0.162 (0.088)	−0.190 (0.101)	−0.173 (0.106)
White Population	0.001 (0.001)	0.002 (0.002)	0.002 (0.002)
Black Population	−0.008 (0.003) *	−0.009 (0.003) *	−0.009 (0.004) *
Hispanic Population	−0.005 (0.002) *	−0.006 (0.003) *	−0.006 (0.003) *
Household Income	0.004 (0.001) ***	0.005 (0.001) ***	0.005 (0.001) ***
Competition	0.003 (0.001) *	0.003 (0.001) *	0.003 (0.001) *
Large Urban Area	−0.150 (0.044) ***	−0.163 (0.051) ***	−0.175 (0.054) ***
Other Urban Area	−0.158 (0.039) ***	−0.173 (0.046) ***	−0.190 (0.048) ***
Mid-Atlantic	−0.281 (0.068) ***	−0.310 (0.079) ***	−0.314 (0.084) ***
East North Central	−0.023 (0.067)	−0.015 (0.078)	−0.038 (0.083)
West North Central	0.016 (0.074)	0.024 (0.086)	0.017 (0.091)
South Atlantic	−0.025 (0.068)	−0.016 (0.078)	−0.037 (0.083)
East South Central	−0.183 (0.075) *	−0.192 (0.088) *	−0.210 (0.092) *
West South Central	−0.194 (0.072) **	−0.208 (0.083) **	−0.234 (0.088) **
Mountain	−0.149 (0.079)	−0.172 (0.092)	−0.184 (0.096)
Pacific	−0.071 (0.072)	−0.087 (0.084)	−0.067 (0.088)
Estimated State Dependence Effect	0.427	0.381	0.341

*** *p* < 0.01, ** *p* < 0.05, * *p* < 0.1, Estimated State Dependence Effect obtained from Equation (8).

**Table 7:** Results of model estimates of marginal effects.

	Marginal Effects	Standard Error	*p* Value
Reward *t* − 1	0.341	0.08	0.004 ***
No. of Employees t − 1	0.043	0.019	0.003 ***
No. of Discharges *t* − 1	−0.003	0.009	0.06 *
Percent of Medicare/Medicaid Discharge *t* − 1	0.009	0.001	0.08 *
Bed Capacity Medium *t* − 1	−0.136	0.074	0.004 ***
Bed Capacity Large *t* − 1	−0.205	0.082	0.008 ***
CMI Q2 *t* − 1	0.014	0.051	0.12
CMI Q3 *t* − 1	0.011	0.024	0.33
CMI Q4 *t* − 1	0.004	0.004	0.16
Government Owned	0.027	0.037	0.46
Voluntary Nonprofit	0.03	0.01	0.004 ***
Very Minor Teaching	−0.08	0.03	0.034 **
Minor Teaching	−0.055	0.013	0.06 *
Major Teaching	−0.113	0.068	0.07 *
Very Major Teaching	−0.142	0.18	0.11
White Population	0.0009	0.0007	0.22
Black Population	−0.0036	0.0017	0.04 **
Hispanic Population	−0.0017	0.0013	0.16
Household Income	0.002	0.001	0.001 ***
Competition	0.001 (0.001) *	0.0008	0.06 *
Large Urban Area	−0.07	0.051	0.001 ***
Other Urban Area	−0.08	0.046	0.002 ***
Mid-Atlantic	−0.14	0.079	0.004 ***
East North Central	−0.013	0.015	0.83
West North Central	0.008	0.086	0.92
South Atlantic	−0.015	0.078	0.88
East South Central	−0.08	0.08	0.88
West South Central	−0.094	0.023	0.04 **
Mountain	−0.079	0.092	0.184
Pacific	−0.031	0.084	0.152

*** *p* < 0.01, ** *p* < 0.05, * *p* < 0.1.

**Table 8:** Results of model estimates of state dependency.

	Pooled Probit	RE Probit	Wooldridge
reward *t* − 1	0.749 (0.053) ***	0.962 (0.061) ***	0.935 (0.069) ***
number of hospitals	1033	838	600
number of observations	6198	5028	3600
estimated state dependence effect	0.297	0.375	0.365
test of equality of coefficient			
chi-2	8.98		
*p* value	0.011		
pairwise comparison	Large urban area/other urban area	Other urban area/rural area	Rural area/large urban area
chi-2	8.84	3.47	4.13
*p* value	0.003 ***	0.06	0.04 **

*** *p* < 0.01, ** *p* < 0.05.

**Table 9:** Comparison of state dependence effect across different ownership.

	Government-Owned Hospitals	Voluntary Non-Profit Hospitals	Proprietary Hospitals
reward *t* − 1	0.648 (0.091) ***	0.953 (0.043) ***	0.752 (0.076) ***
number of hospitals	380	1618	473
number of observations	2280	9708	2838
estimated state dependence effect	0.265	0.371	0.293
test of equality of coefficient			
chi-2	7.34		
*p* value	0.026		
pairwise comparison	G/V	V/P	P/G
chi-2	8.42	6.39	2.23
*p* value	0.001 ***	0.01 *	0.14
test of equality of coefficient			
chi-2	8.98		
*p* value	0.011		
pairwise comparison	Large urban area/other urban area	Other urban area/rural area	Rural area/large urban area
chi-2	8.84	3.47	4.13
*p* value	0.003 ***	0.06	0.04 **

*** *p* < 0.01, ** *p* < 0.05, * *p* < 0.1.

**Table 10:** Estimation of effects of factors on extent of reward/penalty.

	Extent of Reward	Extent of Penalty
No. of Employees *t* − 1	0.04 (0.03) *	−0.02 (0.01) **
No. of Discharges *t* − 1	−0.01 (0.005) ***	0.007 (0.002) ***
Percent of Medicare/Medicaid	−0.4 (0.1) *	−0.02 (0.08)
Bed Capacity Medium *t* − 1	−0.1 (0.03) ***	0.04 (0.04)
Bed Capacity Large *t* − 1	−0.0009 (0.09)	0.07 (0.05)
CMI Q2 *t* − 1	0.1 (0.03) ***	0.008 (0.04)
CMI Q3 *t* − 1	0.2 (0.04) ***	0.03 (0.04)
CMI Q4 *t* − 1	0.2 (0.04) ***	0.08 (0.05)
Government Owned	−0.06 (0.04)	0.05 (0.03)
Voluntary Nonprofit	−0.01 (0.04)	0.02 (0.02)
Very Minor Teaching	−0.01 (0.04)	−0.008 (0.02)
Minor Teaching	0.06 (0.04)	−0.004 (0.02)
Major Teaching	−0.05 (0.06)	−0.02(0.03)
Very Major Teaching	0.04 (0.1)	−0.01 (0.04)
White Population	−0.003 (0.001) ***	−0.0002 (0.001)
Black Population	0.004 (0.005)	0.001 (0.001)
Hispanic Population	−0.0002 (0.003)	0.001 (0.001)
Household Income	0.002 (0.0008) ***	−0.0007(0.0005)
Competition	0.0001(0.001)	−0.002 (0.0008) ***
Large Urban Area	−0.2 (0.04) ***	−0.01 (0.03)
Other Urban Area	−0.1(0.03) ***	−0.009 (0.03)
Mid-Atlantic	−0.1 (0.07) *	0.06(0.04)
East North Central	−0.04 (0.06)	0.02 (0.03)
West North Central	0.01 (0.06)	−0.04 (0.05)
South Atlantic	−0.07 (0.06)	0.02 (0.05)
East South Central	−0.1 (0.07) *	0.04 (0.05)
West South Central	−0.1 (0.06) **	−0.02 (0.05)
Mountain	−0.1(0.07)	−0.04 (0.05)
Pacific	−0.02 (0.07)	−0.01 (0.05)

*** *p* < 0.01, ** *p* < 0.05, * *p* < 0.1.

## Data Availability

The data used to support the findings of this study are available from the corresponding author upon request.
